# Culture conditions for equine bone marrow mesenchymal stem cells and expression of key transcription factors during their differentiation into osteoblasts

**DOI:** 10.1186/2049-1891-4-40

**Published:** 2013-10-29

**Authors:** Elizabeth R A Glynn, Alfredo Sanchez Londono, Steven A Zinn, Thomas A Hoagland, Kristen E Govoni

**Affiliations:** 1 Department of Animal Science, University of Connecticut, 3636 Horsebarn Road Ext., Unit 4040, Storrs, CT 06269-4040, USA; 2 Department of Environmental and Population Health, Tufts University, North Grafton, MA 01536, USA

**Keywords:** Bone marrow mesenchymal stem cells, Cell culture, Equine, Osteoblasts, Transcription factors

## Abstract

**Background:**

The use of equine bone marrow mesenchymal stem cells (BMSC) is a novel method to improve fracture healing in horses. However, additional research is needed to identify optimal culture conditions and to determine the mechanisms involved in regulating BMSC differentiation into osteoblasts. The objectives of the experiments were to determine: 1) if autologous or commercial serum is better for proliferation and differentiation of equine BMSC into osteoblasts, and 2) the expression of key transcription factors during the differentiation of equine BMSC into osteoblasts. Equine BMSC were isolated from the sterna of 3 horses, treated with purchased fetal bovine serum (FBS) or autologous horse serum (HS), and cell proliferation determined. To induce osteoblast differentiation, cells were incubated with L-ascorbic acid-2-phosphate and glycerol-2-phosphate in the presence or absence of human bone morphogenetic protein2 (BMP2), dexamethasone (DEX), or combination of the two. Alkaline phosphatase (ALP) activity, a marker of osteoblast differentiation, was determined by ELISA. Total RNA was isolated from differentiating BMSC between d 0 to 18 to determine expression of *runt-related transcription factor2* (*Runx2*), *osterix* (*Osx*), and *T-box3* (*Tbx3*). Data were analyzed by ANOVA.

**Results:**

Relative to control, FBS and HS increased cell number (133 ± 5 and 116 ± 5%, respectively; *P* < 0.001) and 5-bromo-2'-deoxyuridine (BrdU) incorporation (167 ± 6 and 120 ± 6%, respectively; *P* < 0.001). Treatment with DEX increased ALP activity compared with control (1,638 ± 38%; *P* < 0.001). In the absence and presence of Dex, BMP-2 did not alter ALP activity (*P* > 0.8). *Runt-related transcription factor2* expression increased 3-fold (*P* < 0.001) by d 6 of culture. *Osterix* expression increased 9-fold (*P* < 0.05) by d 18 of culture. Expression of *Tbx3* increased 1.8-fold at d 3 (*P* < 0.01); however expression was reduced 4-fold at d 18 (*P* < 0.01).

**Conclusions:**

Dexamethasone, but not BMP-2, is required for differentiation of equine BMSC into osteoblasts. In addition, expression of *Runx2* and *osterix* increased and expression of *Tbx3* is reduced during differentiation.

## Background

Equine bone fractures are often catastrophic and potentially fatal, as well as costly to owners [1]. It is estimated that annual losses due to bone fractures in the horse-racing industry exceed $10 million [2]. To repair a fracture, costs vary greatly between $2,000 and $10,000 per surgery [3]. This does not include the post-surgical care and rehabilitation which can cost between $100 and $2,000 per d for the first 3 to 4 wk after the surgery [4,5]. Traditional methods of healing fractures have limited success, leading to the need for additional research to identify more successful and economical ways of healing fractured bone [6,7]. Recent advancements include the use of adult stem cells, specifically, bone marrow mesenchymal stem cells (BMSC), in fracture healing [8]. However, the methods for isolation and culture of these cells, and mechanisms regulating BMSC differentiation into osteoblasts (bone forming cells) are not well established in the equine model. Research utilizing rodent or human models has demonstrated that BMSC have the ability to improve bone healing while reducing the risk of re-fracture [9,10] and that addition of certain factors [i.e., fibroblast growth factor, bone morphogenic protein (BMP)] improve fracture healing. Before the findings in humans and rodents can be translated to horses, it must be determined if equine BMSC function similar in culture and if similar mechanisms are involved in regulating the differentiation of BMSC into osteoblasts.

Several studies have attempted to determine the optimal culture conditions for BMSC in various species since expansion of these cells is often needed before reintroduction to the fracture site. The source of serum can affect cell function; however the effects of autologous vs. commercially available serum are variable. Specifically, in rats, BMSC grown in autologous serum had greater growth rates and larger colony sizes than BMSC grown in fetal bovine serum (FBS) [11]. In human BMSC, it was determined that FBS vs. autologous serum did not affect cell number doubling or deoxyribonucleic acid (DNA) copy number [12]. In the equine model, there is evidence that BMSC grow slower in autologous serum compared with FBS [13], but results are often variable depending on the study conditions and objectives. Based on the discrepancy between experiments and species, further experiments are needed to determine the source of serum for culturing BMSC for use either as therapeutic agents or for *in vitro* experiments.

Through human and rodent models we know that the differentiation of BMSC into osteoblasts is a complex process that is regulated by a number of transcription factors. In particular, there are two master genes, *runt-related transcription factor 2* (*Runx2*; required for early differentiation) and *osterix* (*Osx*; required for later differentiation), required for osteoblast differentiation and mineralization [14]. Characterization of these transcription factors and effects of certain agents (dexamethasone, BMP) on differentiation of BMSC into osteoblasts are not well known in the equine model. Another transcription factor recently identified to have a role in osteoblast differentiation is *T-box3* (*Tbx3*). Over-expression of Tbx3 inhibits osteoblast differentiation and the expression of *Runx2* and *Osx* in mouse osteoblasts [15]; however, the expression of Tbx3 has not been characterized in the horse. Therefore, the objectives of the current research were to determine: 1) if autologous or commercial serum is better for proliferation and differentiation of equine BMSC into osteoblasts, and 2) the expression of key transcription factors (*Runx2, Osx, Tbx3*) during the differentiation of equine BMSC into osteoblasts.

## Material and methods

Before the start of the experiments protocols were approved by the Institutional Animal Care and Use Committee at the University of Connecticut.

### Animals

Morgan horses (N = 3; 1 male, 2 females) between 3 and 5 yr of age from the University of Connecticut herd were used. A complete physical examination of the animals was conducted by a veterinarian before procedures.

### Serum collection for cell culture

Serum (HS) was collected from each animal one wk before BMSC isolation. Animals were sedated with detomidine hydrochloride (0.02 mg/kg btw) intravenously. Approximately 500 mL of blood was taken and and stored in 50 mL tubes. Blood was incubated at room temperature for 2 to 4 h to allow clotting. The blood was refrigerated at 4°C for 10 to 12 h. Next, the tubes were centrifuged at 1,800 × *g* for 30 min at 4°C. In a sterile environment, the serum was transferred by gently pipetting into a new tube. The serum was filtered through 0.22 μm pores. Next, the serum was frozen at -20°C for 12 h. The next d, the serum was thawed to room temperature, heat inactivated by incubating at 56°C for 30 min, filtered through 0.22 μm pores, and stored at 4°C for immediate use or -20°C for future use in cell culture experiments [13].

### Bone marrow aspiration

Approximately 1.5 mL of 2% lidocaine were used to infiltrate the subcutaneous area directly above a jugular vein. A 14 gauge, 6 inch catheter was inserted and the horse was sedated with detomidine hydrochloride (0.02 mg/kg body weight) and butorphanol tartrate (0.1 mg/kg body weight). The area of the sternum was clipped and prepared aseptically. Approximately 8 mL of 2% lidocaine was infiltrated into the subcutaneous tissue, muscle, and periosteum. A Jor-Vet Bone Marrow biopsy needle (Jorgensen Laboratories, Loveland, CO, USA) was introduced through a stab incision, made in the soft tissue area of the sternum, and advanced through the muscle layers until it made contact with the ventral surface of the sternum [16]. Pressure was applied to advance the needle until it was seated 1 to 2 cm into the bone. A total of 5 to 15 mL of bone marrow were obtained from each horse using a 20 mL syringe containing 5 mL heparin. The incision was closed with one to two surgical staples. Following bone marrow aspiration, horses received flunixin meglumine (1.1 mg/kg body weight) daily intravenously, twice for the first 3 d and then once daily for d 4 through 6. Staples were removed 14 d after procedure and no negative effects of the procedures were observed in donor horses.

### Culture of BMSC

The bone marrow was placed on ice, transported to the laboratory (approximately 1 km), transferred to a 25 mL canonical tube, and centrifuged at 1,000 × *g* (3 min; room temperature). The supernatant was removed and pellet re-suspended in 10 mL α-minimum essential medium (MEM; Life Technologies, Grand Island, NY, USA) and 40 mL ammonium chloride (Stemcell technologies, Vancouver, BC, Canada) to lyse red blood cells. The sample was vortexed, placed on ice for 10 min, and re-centrifuged at 1,000 × *g* (3 min; room temperature). The supernatant was removed, cells were resuspended in 10 mL α-MEM, and vortexed. The sample was filtered (70 μm cell strainer), reconstituted in maintenance media (Table 1), and plated in a 100 mm cell culture dish (1 per 2 mL of bone marrow). In preliminary experiments with these cells, we determined that some cells attached quickly (within 1 h) and other cells took 3 to 7 d to adhere to the culture dish. To determine if there was a difference in the ability of these cells to proliferate, cells were separated for initial proliferation experiments. The cells were incubated at 37°C, 5% CO_2_ for 20 min to establish the early adherence cell population. Six milliliters of maintenance media were added to adherent cells. Non-adherent cells were removed, counted and re-plated at 6 or 12 million cells/plate in maintenance media to establish the late adherent population. All cells were maintained at 37°C, 5% CO_2_ for 3 d and then 4 mL fresh maintenance media added. After 6 d, media and any remaining non-adherent cells were removed, and cells washed with PBS and media replaced every 3 d until cells reached 70% confluence. All experiments were repeated two to four times using cells from each of the three animals at the fourth passage. Results were consistent across animals and replicates.

**Table 1:** **Cell culture media**^**1**^

**Media type**	**Composition of media**
Maintenance media	α-MEM + 10% FBS + 1% PS + 1% AB
Proliferation media	α-MEM + 0.1% BSA + 1% PS + 1% AB
ALP media	α-MEM + 0.1% BSA + 1% PS + 1% AB + VC + BG
Differentiation media	α-MEM + 10% FBS + 1% PS + 1% AB + VC **+** BG + DEX

^1^ALP = alkaline phosphatase; AB = amphotericin B; α-MEM = α-minimum essential medium; BSA = bovine serum albumin; BG = glycerol 2-phosphate disodium salt hydrate; DEX = dexamethasone; FBS = fetal bovine serum; PS = penicillin/streptomycin concentrate; VC = L-ascorbic acid 2-phosphate sesquimagnesium salt.

### Cell proliferation

Cell proliferation was determined by alamarBlue and 5-bromo-2'-deoxyuridine (BrdU) assays [15]. Cells were passed and plated in a 96-well plate (3,000 cells/100 μL maintenance media/well; n = 8 wells/treatment) and allowed to adhere for 48 h. Cells were serum deprived for 24 h and treated with or without 5% FBS (Stemcell technologies) or 5% HS for 48 h. For alamarBlue assays, cells were rinsed twice (PBS), and a 1:10 dilution of alamarBlue indicator (Life Technologies): α-MEM at 100 μL/well was added. Plates were incubated for 4 h at 37°C, 5% CO_2_ and fluorescence was detected (Synergy2; Biotek, Winooski, VT, USA) at 540/35 × 600/40 nm with sensitivity at 54. For the BrdUrd assay, the Cell Proliferation ELISA BrdUrd (chemiluminescent) Kit (Roche Diagnostics, Indianapolis, IN, USA) was used according to the manufacturer’s protocol.

### Cell differentiation

To determine the ideal reagents to use to stimulate osteoblast differentiation, alkaline phosphatase (ALP) enzyme activity was determined as an endpoint [17]. This is a well-established early marker of osteoblast differentiation in *in vitro* experiments. Cells were passed and plated in a 96-well plate (6,000 cells/100 μL maintenance media/well; n = 8 wells/treatment). After 48 h, cells were rinsed twice and re-plated in serum-free ALP media (Table 1). After 24 h, cells were treated in the absence or presence of human BMP-2 (30 or 60 ng/mL; PeproTech, Rocky Hill, NJ, USA), dexamethasone (DEX; 25 mg/mL; Sigma Aldrich, St. Louis, MO, USA), or the combination of the two [18]. After 72 h, ALP activity was determined as previously described [19] and standardized to protein (Quick Start Bradford Protein; Bio-Rad, Hercules, CA, USA). Specifically, cells were washed twice with PBS and permealized with 100 μL of 0.1% triton, followed by a freeze and thaw. Of the total 100 μL, 40 μL and 20 μL of the lysate were transferred to two new 96-well plates for determination the ALP activity and protein concentration, respectively. To determine ALP activity, 200 μL of the ALP substrate, p-Nitrophenyl phosphate (Sigma), were added to each well. The absorbance was read immediately (0 hr) and kinetically every 10 min for approximately 2 h after substrate addition at 405 nm using the plate reader (Synergy 2; Biotek). The protein concentration was determined by measuring the absorbance following treatment with Quick Start Bradford Protein (Bio-Rad). The protein concentration was standardized with serial dilutions of bovine serum albumin (BSA; Sigma Aldrich). The ALP activity was standardized to the cellular protein content.

To determine the expression of key markers of osteoblast differentiation and transcription factors involved in regulating osteoblast differentiation, we cultured cells in differentiation medium for 18 d. Based on our findings that DEX, but not BMP-2, stimulated ALP activity, we used DEX for our gene expression experiments. Equine BMSC were plated in 6-well plates (150,000 cells/plate/2 mL maintenance media). At 90% confluence, the media were changed to differentiation media (d 0; Table 1), new media were replaced every 3 d, and cells were stained on d 0 and 18 with Alizarin Red and ALP to confirm osteoblast differentiation during the 18 d of culture as previously described [15,19]. Briefly, for Alizarin Red Staining (Sigma Aldrich), media were removed, and then cells were fixed with cold methanol and frozen at -20°C (12 h). Cells were then stained with Alizarin Red, and incubated on a rotator (10 min; room temperature; 100 rpm). Stain was removed and plates were air-dried. For ALP staining, media were removed and cells fixed in cold 100% methanol (20 min; room temperature). Methanol was removed, substrate-diazonium solution added and cells were incubated (30 min; 37°C). Then stain was removed and plates were stored (4°C). Photographs of each well were taken (Olympus IX70; Olympus, Center Valley, PA, USA), and ALP and Alizarin Red stains were quantified from each photograph by determining the amount of stain incorporated using ImageJ software. Data are expressed as a percentage of cells stained on d 0.

### Gene expression analysis

As described in the previous section, cells were plated in 6-well plates (150,000 cells/well) and at 90% confluence, media were changed to differentiation media (d 0), for 18 d and RNA was extracted every 3 d [20] utilizing TriReagent (Sigma) and RNeasy Mini Kit (Qiagen, Valencia, CA, USA) according to the manufacturer’s protocol. Contaminating DNA was removed (TURBO DNA-free kit; Life Technologies), RNA quality determined (Experion RNA StdSens Analysis; Bio-Rad) and concentration determined (NanoDrop spectrophotometer; Fisher Scientific, Pittsburgh, PA, USA).

Gene expression was determined by real-time reverse transcriptase (RT)-PCR as previously described [20] using Superscript II (Invitrogen, Grand Island, NY, USA) and 7900HT Fast Real-Time PCR system (Life Technologies). Each reaction contained 10 μL of 2X Power SYBR Green Master Mix (Life Technologies), 1 μL of 10 mmol/L of forward and reverse primer (Table 2), 3 μL of nuclease free water, and 5 μL of cDNA. *Glyceraldehyde 3-phosphate dehydrogenase* (*GAPDH*) was used as an endogenous control. DeltaCT values were determined and comparisons of the deltaCT values were used for relative quantification [21]. Expression of *osteocalcin* (*Oc*) and *ALP*, key markers of osteoblast differentiation, were determined to confirm differentiation into osteoblasts (Table 2).

**Table 2:** **Primer sequences for real-time reverse transcription-PCR**^**1**^

**Gene**	**(5’–3’)**	**Accession number**^**2**^
ALP (Forward)	GACATGACCTCCCAGGAAGA	XM_001504312.1
ALP (Reverse)	GCAGTGAAGGGCTTCTTGTC	
GAPDH (Forward)	ATCACTGCCACCCAGAAGAC	NM_001163856
GAPDH (Reverse)	GTGAGCTTCCCATTCAGCTC	
Osteocalcin (Forward)	GTGCAGAGTCTGGCAGAGGT	XM_001915727.1
Osteocalcin (Reverse)	CCAGCCAATGATCCAGGTAG	
Osterix (Forward)	GCTCACTATGGCTCCAGTCC	XM_001494930.3
Osterix (Reverse)	AAGGTCACTGCCCACAGAGT	
Runx2 (Forward)	CAGACCAGCAGCACTCCATA	XM_001502519.3
Runx2 (Reverse)	GCAGCATTCTGGAAGGAGAC	
Type I Collagen (Forward)	TTGACCCTAACCAAGGATGC	AB070840.1
Type I Collagen (Reverse)	TTCTTGGCTGGGATGTTTTC	
T-box3 (Forward)	GCATCCCTTTCTCGTCTCTG	XM_001914978
T-box3 (Reverse)	GACCATCTCGGTACCCCTCT	

^1^ALP = alkaline phosphatase; GAPDH = glyceraldehyde 3-phosphate dehydrogenase.

Runx2 = runt-related transcription factor 2.

^2^All sequences from NCBI.

### Statistical analysis

Data were analyzed using the PROC-MIXED procedure of the statistical analysis software (SAS Inst. Inc, Cary, NC, USA) with animal within treatment as the random statement. Suitable corrections were made for multiple comparisons. Differences between means were determined using LSMEANS where appropriate. Data are presented as mean ± SEM with significant difference at *P* ≤ 0.05.

## Results

### Conditions for proliferating equine BMSC

**Figure 1:**
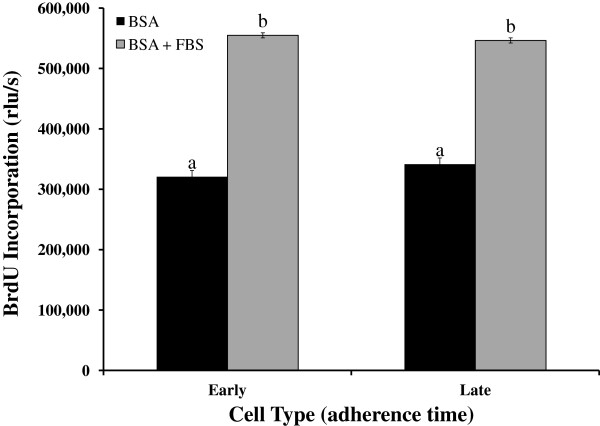
**Proliferation is similar between early and late adherence equine bone marrow stromal cells.** Cells were cultured in the absence [bovine serum albumin (BSA)] or presence (BSA + fetal bovine serum) of serum and proliferation was determined by alamarBlue and 5-bromo-2'-deoxyuridine (BrdU) assays. Data presented are from the BrdU assay and similar findings were observed with alamarBlue assay. Data are presented as mean ± SEM. Bars with different letters are significantly different (*P* < 0.05). rfu/s = relative fluorescence units/s.

To determine if BMSC that adhere early (within 20 min) or late (within 6 d) differ in their proliferation rate, we determined cell number and BrdU incorporation. In the absence and presence of serum (FBS), we did not observe a difference in proliferation of cells between early and late adherence groups (*P* > 0.60) as determined by BrdU incorporation (Figure 1). In both cell populations FBS increased cell proliferation compared with serum-free media (BSA; *P* < 0.0001) as demonstrated by BrdU incorporation (Figure 1). Similarly, in the alamarBlue assay, FBS stimulated cell proliferation in both early and late adherent cells, but we did not detect a difference in their response to FBS (134 ± 5 vs. 133 ± 5% of control for early and late, respectively; *P* > 0.20).

**Figure 2:**
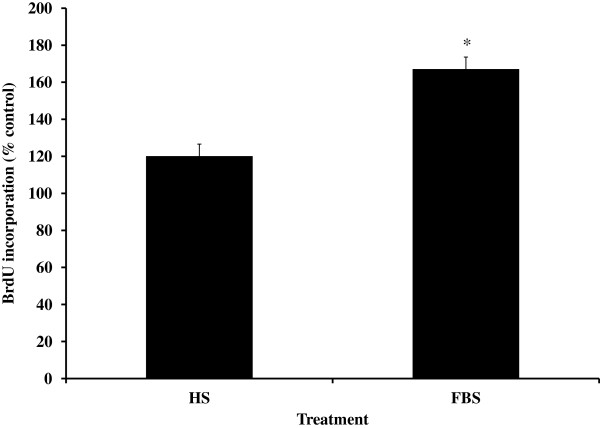
**Cell proliferation is greater in the presence of fetal bovine serum (FBS) compared with horse serum (HS).** Equine bone marrow stromal cells were treated with 5% HS or 5% FBS and cell proliferation was determined by alamarBlue and 5-bromo-2'-deoxyuridine (BrdU) assays. Data are presented from the BrdU assay and similar findings were observed with the alamarBlue assay. Data are presented as mean ± SEM and expressed as a % of control. *indicates a significant difference between the two treatments at *P* < 0.02.

To determine if autologous serum provided a better environment for proliferation than commercially available FBS, cells were cultured in the presence or absence of HS or FBS. Both HS and FBS significantly increased BrdU incorporation (Figure 2; *P* < 0.05); however cells treated with FBS had a greater increase in BrdU incorporation (Figure 2) than cells treated with HS (*P* < 0.02). Similar findings were observed with alamarBlue assay (116 ± 5 vs. 133 ± 5% compared with control for HS vs. FBS, respectively; *P* = 0.01). Based on these data, we used the late adherent cells with FBS supplement for the remaining experiments.

### Effects of BMP-2 and DEX on differentiation of BMSC into osteoblasts

**Figure 3:**
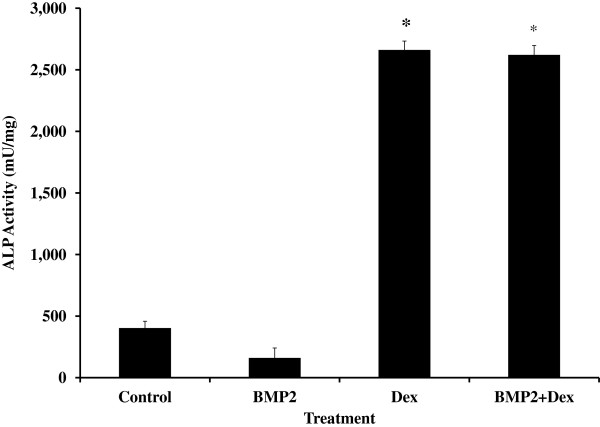
**Dexamethasone (DEX) increases alkaline phosphatase (ALP) activity in equine bone marrow stem cells.** Cells were cultured in the absence (Control) or presence of DEX and bone morphogenic protein 2 (BMP2; 60 ng/mL) and differentiation was determined by ALP enzyme activity. Data are presented as mean ± SEM. *indicates a significant difference compared with control (*P* < 0.0001).

Cells were treated with human BMP-2 and DEX, known stimulators of differentiation of BMSC into osteoblasts. Dexamethasone increased ALP activity 6-fold (*P* < 0.0001; Figure 3). In both the absence and presence of DEX, BMP-2 did not alter ALP activity (No Dex: 150 ± 88, 143 ± 81, and 160 ± 81 mU/mg for 0, 30, and 60 ng/mL hBMP-2; With Dex: 2,661 ± 57, 2,310 ± 76, and 2,621 ± 76 mU/mg for 0, 30, and 60 ng/mL hBMP-2; *P* > 0.8; Figure 3).

### Gene expression during differentiation of BMSC into osteoblasts

**Figure 4:**
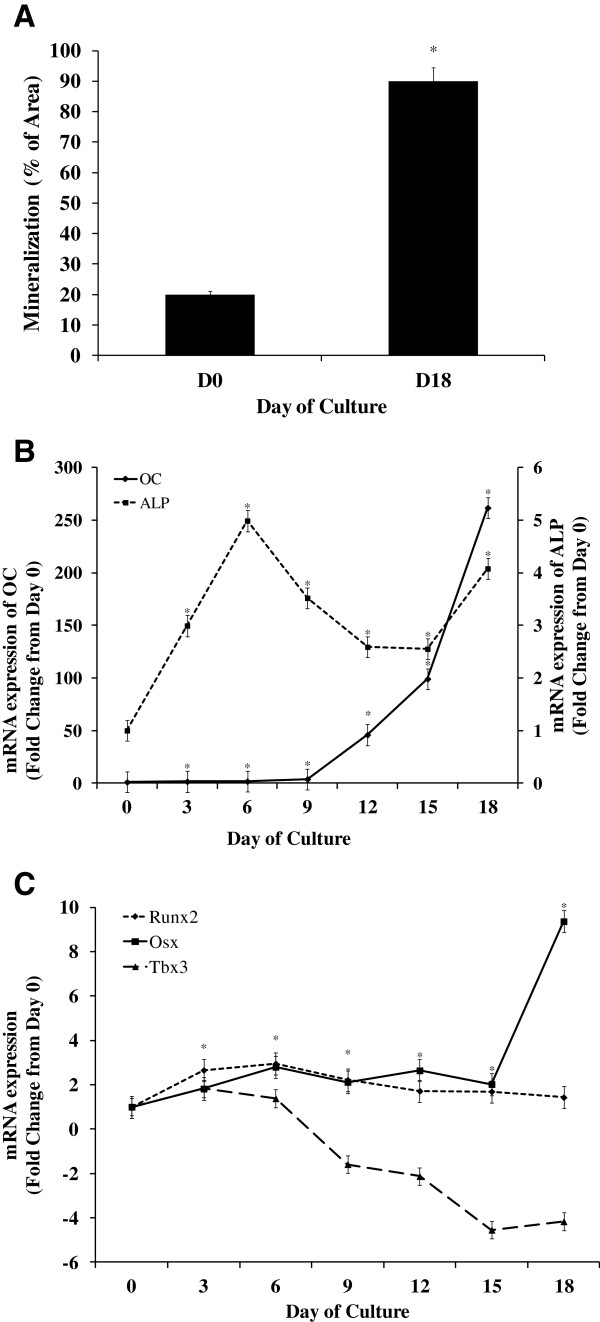
**Expression of key transcription factors during differentiation of equine bone marrow stem cells into osteoblasts.** Equine bone marrow stromal cells were treated with differentiation media for 18 d. **A**. Cells were stained with Alizarin Red on d 0 (DO) and 18 (D18). Quantification of mineralization is presented as mean ± SEM. *indicates a significant difference between d 0 and 18 (*P* < 0.05). Real-time reverse transcription PCR was performed on samples every 3 d (n = 6 wells/d) to determine expression of: **B**. markers of osteoblast differentiation [*osteocalcin* (*OC*) and *alkaline phosphatase* (*ALP*)] and **C**. key transcription factors [*osterix* (*Osx*), *runt-related transcription factor 2* (*Runx2*) and *T-box3* (*Tbx3*)]. *Glyceraldehyde 3-phosphate dehydrogenase* (*GAPDH*) was used as the endogenous control. Data are presented as fold change from d 0 (mean ± SEM). *indicates that a significant difference in mRNA expression from d 0 was observed for all genes on all d, except for Tbx3 expression on d 6 (*P* < 0.05).

By d 18 of culture a 4-fold increase in Alizarin Red stain was observed compared with d 0 (*P* < 0.05; Figure 4A), demonstrating successful differentiation into osteoblasts. Consistent with these findings, a 50% increase in ALP staining was observed between d 0 and 18 (*P* < 0.05).

During differentiation, *ALP* mRNA expression increased by d 6 and remained elevated, although variable, through d 18 of culture compared with d 0 (*P* < 0.001; Figure 4B). In addition, *osteocalcin* expression increased over 200-fold by d 18 of culture compared with d 0 (*P* ≤ 0.05; Figure 4B), thus confirming the differentiation of BMSC into osteoblasts.

*Runt-related transcription factor 2* expression increased 3-fold on d 6 and remained increased through d 18 of culture compared with d 0 (*P* < 0.01; Figure 4C). *Osterix* expression increased as early as d 6 and by 9-fold at d 18 compared with d 0 (*P* < 0.05; Figure 4C). In addition, expression of *Tbx3*, increased 1.8-fold at d 3 (*P* = 0.05), but then decreased more than 4-fold by d 15 of culture compared with d 0 (*P* < 0.05; Figure 4C).

## Discussion

Similar to previous work using equine BMSC in culture, equine BMSC were isolated and able to proliferate and differentiate using standard culture media and serum [22,23]. At the onset of these cell culture experiments with BMSC, it was determined that there are two populations of cells that adhere to the plastic culture dish at different rates. A recent publication demonstrated that these two populations differ in adherence rate, but still retained their capacity to differentiate along the osteogenic lineage [24], therefore we further evaluated the ability of these cells to attach and proliferate in our culture system. The current findings demonstrate that these two populations of cells do not differ in their proliferative rate which is similar to findings in a human model [24]. Based on the findings that BMSC from both early and late populations had a similar proliferative rate, it was concluded that either population of cells could be used for additional experiments, and future experiments should consider using a combination of the two populations.

The use of autologous serum for reintroduction of stem cells is advantageous to reduce rate of rejection. However, the benefits of using autologous serum in culturing BMSC for expansion and *in vitro* experiments are not clear. Based on conflicting reports in rodent and equine models [11,13], the effects of HS vs. FBS on cell proliferation were determined. The findings that FBS had a greater stimulatory effect on cell proliferation than autologous serum are consistent with previous reports in the equine model [13], but are in contrast to findings in a rat model [11]. The differences observed between HS and FBS may be due to the older age of the horses from which serum was collected compared with the young age of the cattle used for commercial serum collection. In addition, commercially produced FBS may contain less factors (i.e., bacteria) that could inhibit proliferation than the autologous HS due to the various steps used to purify commercial serum vs. the basic filtration protocols used in most research laboratories for autologous serum. Based on the consistent findings in the current and previous research, it was concluded that for *in vitro* experiments, BMSC should be cultured in FBS for optimal *in vitro* proliferation which is critical for rapid development of a pool of BMSC. In addition, the use of FBS is advantageous because FBS is a commercially available product of good quality which will provide more consistency in research performed in different laboratories. It should be noted that proliferation experiments were for a short duration (48 h) and additional experiments are needed to determine if similar effects are observed when cells are cultured for several wk or multiple passages. We did observe during our experiments that cells were able to be passed several times (10 or more) without noticeable differences in cell proliferation or viability.

To determine the expression of key transcription factors known to regulate differentiation of BMSC into osteoblasts, the first step was to determine factors that would stimulate osteoblast differentiation, such as dexamethasone and BMP [25,26]. Consistent with findings in rodents and humans [27], DEX increased the differentiation of BMSC into osteoblasts. An effect of BMP-2 on the differentiation of BMSC into osteoblasts was not observed which is in contrast to a recent report in the equine model in which BMP-2 stimulated differentiation of BMSC [19]. Carpenter et al. used a retroviral vector to introduce human BMP-2 [19], whereas we added it directly to the media at one time point. These findings suggest that the method of addition of BMP-2, dose, and duration of treatment may have an impact on the efficacy of BMP-2 treatment. Further studies are needed to determine if continuous treatment with BMP-2, as seen with transduction of the gene, is more effective than a single dose in culture.

Similar to previous reports in rodents and humans expression of *ALP* and *osteocalcin* increased during the differentiation of equine BMSC into osteoblasts [15,28]. *Alkaline phosphatase*, an early marker of osteoblast differentiation, rapidly increased by d 3 and 6, whereas expression was variable at later time points. It is not clear what contributed to the variation, but important to note that expression remained elevated while expression of *osteocalcin*, a later marker of differentiation, continued to increase greatly through d 18. These findings confirm the effectiveness of the differentiation media and that equine BMSC can be induced to differentiate along the osteoblast lineage in culture.

Expression of key master regulatory transcription factors, *Runx2* and *Osx*, was similar to rodents and humans, such that expression of *Runx2* was greatest during the first 6 d of differentiation and *Osx* expression was greatest at the final d of culture [28]. These findings suggest that similar to other species, *Runx2* and *Osx* may be critical for optimal bone formation, mineralization and osteoblast function in horses [29]. The methods used in the current study to induce differentiation of BMSC into osteoblasts were successful and equine osteoblast differentiation may be regulated similarly to osteoblast differentiation in other species. Further studies are needed using knockdown of these transcription factors to determine their specific roles in osteogenesis in the equine model. Since *Runx2* and *Osx* are known to be expressed during early and late differentiation, respectively [29], the current findings will be helpful in future studies that aim to determine the effectiveness of specific factors in inducing the differentiation of equine BMSC into osteoblasts *in vitro*.

In the mouse, *Tbx3* was recently identified as an important stimulator of osteoblast proliferation and inhibitor of differentiation, as well as responsive to growth hormone [15,20]. We provide evidence that *Tbx3* is expressed in equine BMSC and its expression changes with differentiation along the osteoblast lineage. The reduction in *Tbx3* expression as BMSC differentiate into osteoblasts is consistent with a mouse *in vitro* model, in which over-expression of *Tbx3* represses differentiation of BMSC into osteoblasts [15]. These findings suggest that *Tbx3* may need to be suppressed for optimal differentiation. More importantly, in addition to demonstrating that expression of key transcription factors during osteoblast differentiation in horses are similar to human and rodent models, these experiments provide novel data that *Tbx3* expression decreased during differentiation.

## Conclusions

In conclusion, the results of the current study demonstrate greater proliferation of equine BMSC using a commercially available serum and that expression of *Runx2* and *Osx* during differentiation is similar to those reported in other species. In addition, we provide the first evidence that *Tbx3* is expressed in equine cells and expression is reduced during differentiation, suggesting a potential inhibitory role in differentiation. Overall, these findings broaden our knowledge of culture conditions for equine BMSC and expression of key regulatory factors during differentiation. These findings will help to improve culture conditions of equine BMSC for future experiments that aim to utilize these cells for fracture healing in horses.

## Abbreviations

ALP: Alkaline phosphatase; BMSC: Bone marrow mesenchymal stem cells; BMP: Bone morphogenic protein; BrdU: 5-bromo-2'-deoxyuridine; DNA: Deoxyribonucleic acid; DEX: Dexamethasone; E: Equine; FBS: Fetal bovine serum; HS: Horse serum; Osx: Osterix; Runx: Runt related transcription factor; Tbx: T-box.

## Competing interests

The authors do not have any competing interests.

## Authors’ contributions

ERAG cared for the animals, isolated and cultured cells, performed all *in vitro* experiments and data analysis and assisted in drafting the manuscript. ASL performed all the bone marrow biopsies, cared for the animals and assisted in protocol design. SAZ assisted with experimental design and drafting the manuscript. TAH assisted in experimental design and statistical analysis. KEG conceived of the study, participated in design, sample collection, data analysis and drafting the manuscript. All authors read and approved the final manuscript.
